# De novo transcriptome analysis of a medicinal fungi *Phellinus linteus* and identification of SSR markers

**DOI:** 10.1080/13102818.2015.1008228

**Published:** 2015-02-05

**Authors:** Yating Huang, Xiaoqiu Wu, Duan Jian, Yaguang Zhan, Guizhi Fan

**Affiliations:** ^a^ Department of Forest Bioengineering, College of Life Science, Northeast Forestry University, Harbin, P.R. China

**Keywords:** *Phellinus linteus*, transcriptome, RNA-sequencing, simple sequence repeat (SSR)

## Abstract

The aim of this study was to facilitate gene discovery for functional genome studies and to identify simple sequence repeat (SSR) markers for molecular-assisted selection in *Phellinus linteus*. The transcriptome of *Phellinus linteus* was sequenced using а high-throughput RNA sequencing system – the Illumina Hiseq 2000. A total of 16,383,818 clean sequencing reads, 35,532 contigs and 25,811 unigenes were postulated. Based on similarity searches with known proteins, 19,350 genes (74.97% of the unigenes) were annotated. In the present research, 19,266, 10,978 and 7831 unigenes were mapped in Nr, Swiss-Prot and clusters of orthologous groups (COG) classifications, respectively. Of all unigenes, 6845 were categorized into three functional groups, namely biological process, cellular components and molecular function and 11,088 were annotated to 108 pathways by searching the Kyoto Encyclopedia of Genes and Genomes pathway database. A total of 1129 SSRs were identified in these unigenes. In addition, 23 candidate genes, potentially involved in sterol biosynthesis, were identified and were worthy of further investigation.

## Introduction

*Phellinus linteus*, a Basidiomycete fungus, belonging to the genus *Phellinus*, is one of the most famous traditional Chinese medicines.[1] Its fruiting body is called ‘Sanghuang’ in China. *Phellinus linteus* has been well known as a medically potent mushroom due to its ability to treat various conditions, including gastrointestinal disorders, peptic ulcers, neurodegenerative diseases, lymphatic diseases and various cancers.[2–5] This fungus also has antioxidative, anti-inflammatory and antimutagenic activities. As a result of its perceived health benefits, *Phellinus linteus* has gained a wide popularity as an effective medicine and has become one of the most valuable mushrooms in China.[6]

Currently, commercial products from medicinal mushrooms are mostly obtained through the field-cultivation of the fruiting body.[7–9] However, solid culture does not guarantee a standardized product and in this way the composition of the product may vary from batch to batch.[10] Accordingly, mushroom submerged fermentation may be viewed as a promising alternative for the efficient production of their valuable products.[1] To achieve higher yield in a submerged culture, it is a prerequisite to design an optimal production medium, to set an optimal process operating conditions and to regulate metabolic pathways through biotechnological approaches.[11] The approaches for improving the production of the desired useful metabolites by mushrooms require a prior knowledge of the metabolic pathways and the biosynthesis and genes involved in the metabolic, regulatory and other cellular processes. However, because of the absence of genome information, no gene regulatory study has been reported to date for *Phellinus linteus*.

Transcriptome sequencing is an efficient method for acquiring fungal functional genomics information. In recent years, next generation sequencing techniques, such as Illumina, Roche 454 and SOLID (ABI) platforms, have emerged as useful tools for transcriptome analysis. These tools have been widely used in detecting the gene expression, discovering the novel transcripts, testing the differently expressed genes and gaining other useful information.[12,13] In general, Illumina-based de novo transcriptome sequencing is suitable for species without prior genomic information, such as *Salvia miltiorrhiza*, *Amanita exitialis* and *Alexandrium catenella*.[13,14] Despite its obvious potential, next generation sequencing methods have not yet been applied to *Phellinus linteus* research.

In this paper, we report on and discuss the results of transcriptome sequencing, a search for simple sequence repeat (SSR) and putative unigenes involved in sterol biosynthesis in the *Phellinus linteus*. The transcriptome data, generated from our study, comprise a useful resource for gene excavation and transcriptomic assembly, as well as molecular marker, microarray and biosynthetic pathway development. Additionally, the SSR markers, identified in this study, will contribute to marker-assisted breeding selection, facilitate gene mapping and help with linkage mapping.

## Materials and methods

### Micro-organism and culture conditions

**Figure 1.**
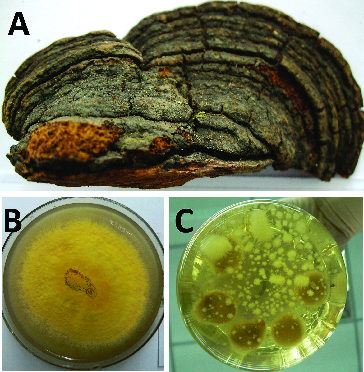
Morphological characteristics of colony and mycelia from *P. linteus* strains. Note: Fruit of *P*. *linteus* from *M. alba* (A), solid colony (B) and liquid mycelia (C).

Mycelia of *Phellinus linteus* were isolated from the fruit of *Morus alba* L, which was found in Changbaishan Mountain in China (Figure 1).[15] *Phellinus linteus* was maintained on potato dextrose agar (PDA) medium, containing 20 g·L^−1^ glucose at 4° C. Liquid cultures were initiated from seven-day-old PDA by inoculating 1.5 cm^2^ agar cubes into 250 mL flask with 80 mL of PDA liquid medium. All media were sterilized at 121° C for 20 min. The Erlenmeyer flasks were incubated on a rotary shaker (110 rpm) at 25 °C for 10 days. Afterwards, the original block of inoculation in the liquid medium was discarded and *Phellinus linteus* mycelia were obtained by filtration after three, six and nine days of cultivation. Cultured mycelia were washed twice with distilled water. The collected samples were then immediately frozen in liquid nitrogen and stored in a −80 °C freezer for later use.

### cDNA library construction and Illumina sequencing

RNA extraction, cDNA library construction and Illumina sequencing of the sample was conducted at BGI-Shenzhen, China (http://www.genomics.cn/index.php), according to the manufacturer's instructions (Illumina, San Diego, CA, USA).[16] Total RNA was extracted from mycelia of *Phellinus linteus* using TRIzol (Invitrogen) and after this, the purity and concentration of the isolated RNA were determined by using Agilent 2100 Bioanalyzer (Agilent Technologies, Santa Clara, CA, USA). RNA samples from cultures from different periods were mixed to prepare an equimolar concentration of total RNA and were used for cDNA library construction. The cDNA was synthesized using the mRNA fragments as templates. Short fragments were purified and resolved with EB buffer for end reparation and single nucleotide A (adenine) addition. The short fragments were then connected with adapters and suitable fragments, which were selected for the PCR amplification as templates. The Agilent 2100 Bioanaylzer and ABI StepOnePlus Real-Time PCR System were used for quantification and qualification of the sample library before sequencing it, using Illumina HiSeq™ 2000.

### Data filtering and de novo assembly

The image data output from the sequencing machine were transformed by base calling into sequence data, which were called raw data or raw reads and stored in FASTQ format. Image deconvolution and quality value calculations were performed using Illumina HCS 1.1 software.[17] The raw reads were cleaned by removing adapter sequences, low-quality sequences (reads with ambiguous bases ‘N’) and reads with more than 10% Q_B20_ bases (those with a base quality value less than 20). Because no reference genome was available for *Phellinus linteus*, we chose the Trinity software to assemble the transcriptome, according to the report by Grabherr et al.[18] Trinity combines three independent software modules: Inchworm, Chrysalis and Butterfly, applied sequentially to process large volumes of RNA-seq reads. The result sequences of the Trinity assembly are called unigenes.

### Functional annotation and Kyoto Encyclopedia of Genes and Genomes (KEGG) pathway analysis

Functional annotation and KEGG pathway analysis were performed according to Zhong et al.[19] The annotation of unigenes was based on sequence homology using BLASTX software. The unigene sequences were searched against the Swiss-Prot database, the Nr database, the KEGG database, the clusters of orthologous groups (COG) database and the NT database (E-value < 1.0 E^−5^). The unique sequences were assigned to special biochemical pathways according to the KEGG standards, using BLASTX. The terms of gene ontology (GO) classification were assigned to all well-annotated sequences by performing Blast2GO program. To reduce the redundancy, each sequence that had BLAST hit in the Nr database was given a unigene ID according to the best homologue they were aligned to.

### Identification of SSR markers

SSR was detected using MIcroSAtellite tool. SSR was detected by considering 100 bp flanking sequences on upstream and downstream of SSR. Parameters used for development of SSR are mentioned in Table 1.

**Table 1.** Parameters used for development of SSR.

No.	SSR type	Set of repeating bases	Repetition number for the set
1.	Mononucleotide	Repeats 1	≥10 bases
2.	Dinucleotide	Repeats 2	≥6 bases
3.	Trinucleotide	Repeats 3	≥5 bases
4.	Tetranucleotide	Repeats 4	≥5 bases
5.	Pentanucleotide	Repeats 5	≥5 bases
6.	Hexanucleotide	Repeats 6	≥5 bases

Note: SSR detection was done with software MicroSAtellite (MISA) using unigenes as reference.

## Results and discussion

### Sequence analysis and de novo assembly

After performing Illumina sequencing, 17,467,362 raw reads were generated (Table 2). Then reads with adaptors, a percentage of unknown nucleotides greater than 5%, or a percentage of low-quality bases (base quality ≤ 10) more than 20%, were filtered out, leaving 16,383,818 clean reads, which were subjected to de novo assembly. The clean reads contained 1,474,543,620 nucleotides with an average length of 90 nucleotides. The values of the Q20 percentage (the proportion of nucleotides with quality value larger than 20 in reads) and N percentage were 99% and 0.01%, respectively. In addition, the GC percentage of the clean reads was 51.49%.

**Table 2.** Statistics of Illumina sequencing.

Total raw reads	17,467,362
Total clean reads	16,383,818
Total clean nucleotides (Nt)	1,474,543,620
Q20 percentage	99.00%
N percentage	0.01%
GC percentage	51.49%

Note: Total raw reads and total clean reads are actually total number of raw reads and total number of clean reads. Total clean nucleotides is the total number of clean nucleotides. Q20 percentage is the proportion of nucleotides with quality value larger than 20 in reads; N percentage is the proportion of unknown nucleotides in clean reads. GC percentage is the proportion of guanidine and cytosine nucleotides among the total nucleotides.

The genome map of *Phellinus linteus* has not been revealed yet; therefore, we chose de novo assembly for the sequencing data (Table 3). As a result, 35,532 contigs were generated, with a total length of 17,841,209 nucleotides and an average length of 502 nucleotides. These contigs were subsequently assembled into 25,811 unigenes. The total length of the unigenes was 25,229,528 nucleotides, and the mean length was 977 nucleotides. Among the unigenes, 9395 were distinct clusters, and the other 16,416 were distinct singletons.

**Table 3.** Statistics of assembly quality.

	Contig	Unigene
Total number	35,532	25,811
Total length (nt)	17,841,209	25,229,528
Mean length (nt)	502	977
N50	1262	1658
Total consensus sequences	−	25,811
Distinct clusters	−	9395
Distinct singletons	−	16,416

Note: Transcriptome de novo assembly is carried out by short reads assembling program -- Trinity. The statistical parameters of the assembly quality in cotig and unigene included total number, total length (nt) and mean length (nt); measure of average contig or unigene length (N50 length), total consensus sequences, distinct clusters and distinct singletons.

In conclusion, all of the statistics illustrated a better quality and depth of the sequencing, produced by Illumina on *Phellinus linteus*.

### Functional annotation of the unigenes

Because of the lack of genetic or genomic information on *Phellinus linteus*, it is difficult to estimate the number of genes and the level of transcript coverage. We performed BLASTX alignments (E-value < 1.0 E^−5^) against the public databases including Nr, NT, Swiss-Prot, KEGG, COG and GO to identify the putative functions of the ALL unigene sequences. A total of 19,350 (74.97% of ALL unigenes) were matched to one or more of the databases (Table 4). Among them, 74.64% match efficiency was observed for sequences in Nr databases, 42.53% in Swiss-Prot databases, 42.96% in KEGG databases and 30.34% in COG databases.

**Table 4.** Summary statistics of functional annotation of *Phellinus linteus* unigenes in public databases.

Public protein database	Number of unigene hits	Percentage (%)
Nr	19,266	74.64
NT	4562	17.67
Swiss-Prot	10,978	42.53
KEGG	11,088	42.96
COG	7831	30.34
GO	6845	26.52
ALL	19,350	74.97

Note: Unigene annotation provides information of the expression and function of unigene. Information of functional annotation gives protein functional annotation, COG functional annotation and gene ontology (GO) functional annotation of Unigenes. Unigene sequences are firstly aligned by BLASTX to protein databases like Nr, Swiss-Prot, KEGG and COG (E-value < 1.0 E^−5^) and aligned by BLASTN to nucleotide databases NT (E-value < 1.0 E^−5^), retrieving proteins with the highest sequence similarity with the given Unigenes along with their protein functional annotations, the results about this are included in the folder annotation.

**Figure 2.**
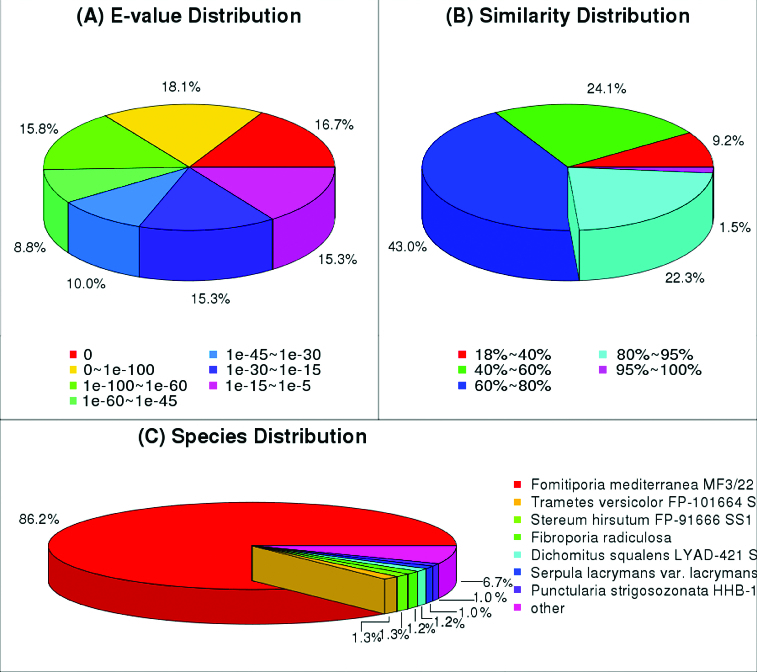
Characteristics of sequence homology of *P. linteus* blasted against NCBI non-redundant (Nr) database. Note: E-value distribution of BLAST hits for matched unigene sequences, using an E-value cutoff of 1.0 E^−45^ (A), similarity distribution of top BLAST hits for each unigene (B) and species distribution of the top BLAST hits (C).

In the alignment with NCBI Nr protein database, 19,266 unigenes were annotated. Among these successfully annotated unigenes, 59.4% had strong homology with the aligned proteins (E-value < 1.0 E^−45^) (Figure 2 (A)). The similarity distribution illustrated that 66.8% of the sequences had a similarity higher than 60% (Figure 2 (B)). As for the species distribution, most of the annotated unigenes were annotated to proteins from *Fomitiporia mediterranea* (86.2%) (Figure 2 (C)).

**Figure 3.**
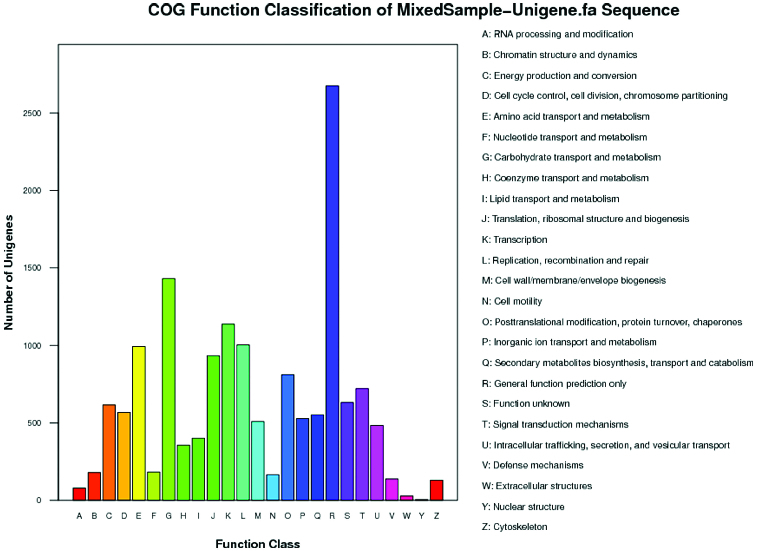
Histogram presentation of clusters of orthologous groups (COG) classification (unigene). Note: All unigenes were aligned to the COG database to predict and classify possible functions. Out of 19,266 NR hits, 7831unigenes were assigned to 25 COG classifications.

To obtain a deeper understanding of the functions of the unigenes, BLASTX alignment was performed between the unigenes and the COG database (Figure 3). The 7831 unigenes were classified into 25 functional categories. Among those categories, the R category (general function prediction only) contained the largest number of unigenes (2675; 17.55%). Other categories that we had interest in were the E category (amino acid transport and metabolism), G category (carbohydrate transport and metabolism), Q category (secondary metabolites biosynthesis, transport and catabolism) and the S category (function unknown). Genes, related to nutritional or medicinal metabolites, were most likely sorted into these four categories. The number of unigenes annotated into the E category, G category, Q category and S category were 992 (6.51%), 1431 (9.39%), 551 (3.62%) and 631 (4.14%), respectively.

**Figure 4.**
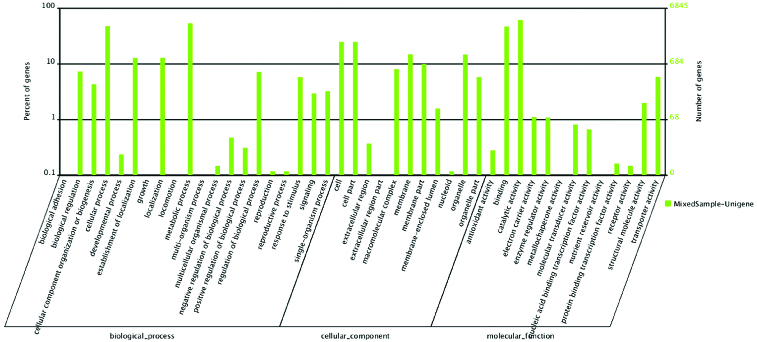
Gene ontology classification of assembled unigenes. Note: The results are summarized in three main categories: biological process, cellular component and molecular function. The right *y*-axis indicates the number of genes in a category. The left *y*-axis indicates the percentage of a specific category of genes.

GO assignments were used to classify the functions of the predicted *Phellinus linteus* genes. Based on sequence homology, 6845 unigenes can be categorized into three functional groups, namely biological process, cellular component and molecular function (Figure 4). Within the molecular function category, catalytic activity (4170, 51.03%) and binding activity (3180, 38.91%) were dominant. For cellular components, most assignments were to cell (1680, 23.62%) and membrane (1004, 14.11%). Within the biological process category, metabolic processes (3611, 33.52%) and cellular processes (3241, 30.08%) were the most highly represented. These annotations indicated that the hyphae of *Phellinus linteus* were undergoing extensive metabolic activity.

The KEGG pathway database deposits the networks of molecular interactions in the cells and is widely used as a reference canonical database for integration and interpretation of large-scale data-set. Therefore, according to KEGG pathway mapping, gene functions with the emphasis on biochemical pathways can be categorized.[20–22]

KEGG database was also searched to obtain information about the biological pathways operating in *Phellinus linteus*. According to the alignment results, 11,088 unigenes were annotated into 108 different pathways in the KEGG database (Table 1S in the Online Supplementary Appendix). The result showed that the five largest pathway groups were metabolic pathways (2875, 25.93%), biosynthesis of secondary metabolites (1227, 11.07%), starch and sucrose metabolism (738, 6.66%), MAPK signalling pathway–yeast (660, 5.95%) and RNA transport (627, 5.65%).

Based on the BLASTX results, we found that a lot of our unigenes matched to known proteins in the four public databases, implying that the Illumina-based sequencing project yielded an extensive and large proportion of the diverse genes expressed in *Phellinus linteus*. These unigenes were assigned a putative gene or protein name descriptions and categorized with GO terms and metabolic pathways because detailed functional information is essential to an overall understanding of the gene expression profiles in *Phellinus linteus*. These annotations provide a valuable resource for investigating specific processes, functions and pathways and will contribute to the identification of novel genes, which are involved in the pathways of secondary metabolite biosynthesis.[23]

### Candidate genes involved in sterol biosynthesis

Sterols are essential lipid constituents in eukaryotic membranes. The structural features of terminal sterols differ among species. Whereas mammalian and fungal cells generally contain one major sterol cholesterol or ergosterol, respectively, plants have complexed sterol profiles, dominated by sitosterol, stigmasterol and campesterol.[24] Ergosterol, produced by fungi, has attracted much attention due to its health properties, such as antioxidant, antiinflammatory, antihyperlipidemic and potential anticancer effects.[25,26] The ergosterol biosynthesis pathway is well characterized in *Saccharomyces cerevisiae*, while little is known about the pathway in filamentous fungi, so we investigated the genes that control sterol biosynthesis in *Phellinus linteus*.

As shown in Table 5, 23 genes, related to sterol biosynthesis of *Phellinus linteus*, were identified. Among them, seven genes belonged to mevalonate pathway in terpenoid backbone biosynthesis. They were all precursor genes, controlling the biosynthesis of ergosterol or phytosterol. Eleven genes belonged to ergosterol biosynthesis pathway and five genes belonged to the phytosterol biosynthesis pathway.

**Table 5.** Unigenes related to sterol biosynthesis in *Phellinus linteus.*

Pathway	Enzyme name	Symbol/gene name	Number of annotated sequences transcripts
Terpenoid backbone biosynthesis	Acetyl-CoA C-acetyltransferase	E.9, atoB	2
	Hydroxymethylglutaryl-CoA synthase	E.10	1
	Hydroxymethylglutaryl-CoA reductase (NADPH)	HMGCR	2
	Phosphomevalonate kinase	mvaK2	2
	Diphosphomevalonate decarboxylase	MVD, mvaD	1
	Isopentenyl-diphosphate delta-isomerase	IDI	1
	Farnesyl diphosphate synthase	FDPS	4
			
Ergosterol biosynthesis	Lanosterol synthase	LSS, ERG7	2
	Sterol 14-demethylase	CYP51	7
	Delta14-sterol reductase	TM7SF2, ERG24	3
	Methylsterol monooxygenase	SC4MOL, ERG25	2
	Sterol-4alpha-carboxylate 3-dehydrogenase (decarboxylating)	NSDHL, ERG26	2
	3-keto steroid reductase	ERG27	2
	Sterol 24-C-methyltransferase	SMT1, ERG6	2
	C-8 sterol isomerase	ERG2	1
	Lathosterol oxidase	ERG3	1
	C-22 sterol desaturase	ERG5	6
	Delta24(24(1))-sterol reductase	ERG4	1
			
Phytosterol biosynthesis	Sterol 24-C-methyltransferase	SMT1, ERG6	2
	Sterol 14-demethylase	CYP51	7
	Delta14-sterol reductase	TM7SF2, ERG24	3
	Cholestenol delta-isomerase	EBP	1
	Lathosterol oxidase	SC, ERG3	1

In general, the fungal ergosterol biosynthesis pathway is divided into mevalonate phase and post-squalene phase. In our study, we found 7 genes in mevalonate pathway and 11 genes controlling the ergosterol biosynthesis. Тhe above-mentioned genes were in accordance with the reported genes in yeast.[24] Furthermore, we also found five genes related to phytosterol biosynthesis. Further identification of the sterol composition profiles of all the available gene regulations of these enzymes will be necessary for a full understanding of the pathway for ergosterol biosynthesis in *Phellinus linteus*. This will be helpful for the large-scale production of medical secondary metabolism.

### Identification of SSRs

Molecular markers play an important role in gene mapping studies and marker-assisted molecular breeding for the improvement of the fungus varieties with the desired traits. Various molecular markers, used for studying these variations, include restriction fragment length polymorphism, random amplified polymorphic DNA, single nucleotide polymorphisms (SNP) and SSR. SSR is one of the most popular marker systems and is consistent by varying numbers of tandemly repeated di-, tri- or tetranucleotides, which are distributed randomly throughout the genome of all eukaryotes.[27]

**Figure 5.**
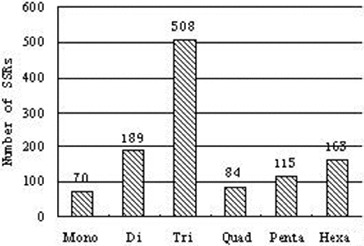
Simple sequence repeats: distribution of SSRs into di-, tri-, tetra-, penta- and hexarepeat types.

Out of the 25,811 sequences that were examined, a total number of 1129 SSRs were identified from *Phellinus linteus.* Statistical analysis of the identified SSRs is presented in Table 6. The number of sequences containing one SSR and more than one SSR were 873 and 177, respectively. The number of mono-, di-, tri-, tetra-, penta- and hexarepeats were 70, 189, 508, 84, 115 and 163, respectively (Figure 5). These SSR will be of immense help in the development of species-specific *Phellinus linteus* markers.

**Table 6.** Statistics of SSRs identified from *Phellinus linteus* transcriptome.

1. Total number of examined sequences	25,811
2. Total size of examined sequences (bp)	25,229,528
3. Total number of identified SSRs	1129
4. Number of sequences containing one SSR	873
5. Number of sequences containing more than one SSR	177
6. Number of SSR present in compound formation	94

## Conclusions

*Phellinus linteus* can be viewed as a potential source of useful metabolites with health-promoting properties. Thus, by using Illumina RNA-Seq technology and de novo analysis, we generated more than 25,811 unigenes with an average of 977 bp in length and 19,350 of the sequences had a significant BLAST hit. We assigned 7831 sequences to 25 COG classifications, 6845 unigenes to three functional groups (biological process, cellular component and molecular function), 11,088 sequences to 108 KEGG pathways and most of the annotated unigenes to proteins from *Fomitiporia mediterranea* (86.2%). A large number of transcript sequences, obtained in this study, were the first representatives of these transcripts for Chinese *Phellinus linteus*. Twenty-three candidate genes, potentially involved in sterol biosynthesis, were identified and were worthy for further investigation. These findings provide a substantial contribution to the existing sequences resources for the *Phellinus linteus* and other filamentous fungi.
